# Conserved miR-370-3p/BMP-7 axis regulates the phenotypic change of human vascular smooth muscle cells

**DOI:** 10.1038/s41598-022-26711-z

**Published:** 2023-02-10

**Authors:** Yerin Kim, Namhee Yu, Ye Eun Jang, Eunkyung Lee, Yeonjoo Jung, Doo Jae Lee, W. Robert Taylor, Hanjoong Jo, Jaesang Kim, Sanghyuk Lee, Sang Won Kang

**Affiliations:** 1 Department of Life Science, Ewha Womans University, Seoul, 03760 Republic of Korea; 2 Research Institute, National Cancer Center, Goyang, 10408 Republic of Korea; 3 Division of Cardiology, Emory University School of Medicine, Atlanta, GA USA; 4 Wallace H. Coulter Department of Biomedical Engineering, Georgia Institute of Technology and Emory University, Atlanta, GA 30322 USA

**Keywords:** Cell biology, Computational biology and bioinformatics

## Abstract

Endothelial dysfunction and inflammatory immune response trigger dedifferentiation of vascular smooth muscle cells (SMCs) from contractile to synthetic phenotype and initiate arterial occlusion. However, the complex vascular remodeling process playing roles in arterial occlusion initiation is largely unknown. We performed bulk sequencing of small and messenger RNAs in a rodent arterial injury model. Bioinformatic data analyses reveal that six miRNAs are overexpressed in injured rat carotids as well as synthetic-type human vascular SMCs. In vitro cell-based assays show that four miRNAs (miR-130b-5p, miR-132-3p, miR-370-3p, and miR-410-3p) distinctly regulate the proliferation of and monocyte adhesion to the vascular SMCs. Individual inhibition of the four selected miRNAs strongly prevents the neointimal hyperplasia in the injured rat carotid arteries. Mechanistically, miR-132-3p and miR-370-3p direct the cell cycle progression, triggering SMC proliferation. Gene ontology analysis of mRNA sequencing data consistently reveal that the miRNA targets include gene clusters that direct proliferation, differentiation, and inflammation. Notably, bone morphogenic protein (BMP)-7 is a prominent target gene of miR-370-3p, and it regulates vascular SMC proliferation in cellular and animal models. Overall, this study first reports that the miR-370-3p/BMP-7 axis determines the vascular SMC phenotype in both rodent and human systems.

## Introduction

Normal arterial vessels have two major cell types, endothelial cell (EC) and smooth muscle cell (SMC). However, the intimal accumulation of low-density lipoproteins combined with disturbed blood flow can inflame the ECs in the monolayer^1^. Generally, monocytes/macrophages attach on the inflamed EC lesion, penetrate the EC monolayer, and take up the lipid particles. In addition to the lipid core, the neutrophil extracellular traps (NETs) cause atherogenesis^2^. The resulting macrophage-driven foam cells and immune cells in the atherogenic lesion secrete many growth factors and cytokines that stimulate SMCs to undergo dedifferentiation and fibrous cap formation. This dedifferentiation process results in the phenotype change from contractile to synthetic type. These atherogenic processes including the inflammatory response and hyperplasia of SMCs eventually cause the arterial lumens to be occluded. Currently, angioplasty with stent procedure is the only method known to repair the occluded arteries in patients with atherosclerotic lesions. However, the major complication post procedure is thrombosis resulting from the endothelial damage and subsequent neointimal hyperplasia^3^, called in-stent restenosis. The restenosis process actually resembles to the late stage of atherogenesis in terms of the proliferation and migration of the dedifferentiated SMCs. Thus, the complex atherogenesis cannot be rescued by any targeted therapy. Since a microRNA (miRNA) named *lin-4* was discovered in the nematode^4^, the function of miRNA as a translational modulator has received fair attention in the past decades. The unique feature of miRNA is to simultaneously modulate the expression of many target genes containing the specific seed sequence at the 3′ untranslated region^5^. Indeed, several groups have attempted to seek and characterize the vascular miRNAs that regulate the SMC growth^6–8^. However, no genome-wide screening of vascular miRNAs has been conducted in human arterial tissues and even in animal models.

Hence, this study aimed to screen vascular miRNAs associated with the arterial SMC hyperplasia in a balloon injury model of rat carotid artery. The small RNA sequencing and data analysis revealed the differential expression of 25 miRNAs. Through cell-based functional validation, four miRNAs that are critical for the inflammatory response and proliferation of arterial SMCs were selected. In particular, the transcriptome analysis by messenger RNA (mRNA) sequencing revealed that the bone morphogenic protein (BMP)-7 is a specific target of miR-370-3p.

## Materials and methods

### Reagents

Anti-SMA antibody (cat. no. 113200) was purchased from Calbiochem. Anti-Calponin antibody (cat. no. HPA014263) was purchased from Atlas antibodies. Anti-p-SMAD1/5/8 (cat. no. 13820T), anti-SMAD1 (cat. no. 9743S), anti-p27 (cat. no. 3688S), and anti-cyclinD1 (cat.no. 2922) antibodies were purchased from Cell Signaling Technology. Anti-BMP-7 antibody (cat. no. ab84684) was purchased from Abcam. Alexa Fluor 488-phalloidin (cat. no. A12379) was purchased from Invitrogen. Cy3-conjugated anti-SMA antibody (cat. no. C6198) and anti-Tubulin antibodies (cat. no. T5168) were purchased from Sigma Aldrich. Anti-p21 antibody (cat. no. sc-6246) was purchased from Santa Cruz Biotechnology. FITC-conjugated anti-von Willebrand factor (vWF) antibody (cat. no. ab8822) was purchased from Abcam. Ad-mBMP-7 (cat. no. ADV-254023) was purchased from Vectorbiolabs. The mirVana miRNA mimics, inhibitors, and negative control were purchased from Invitrogen.

### Balloon-induced injury procedure in rat carotid artery

The study used ten-week-old male Sprague–Dawley rats (Charles River, U.S.A.) for a balloon-induced carotid injury model and is reported in accordance with ARRIVE guidelines. Animal study protocols were approved by the Institutional Animal Care and Use Committee (IACUC) of Ewha Womans University, South Korea, and conformed to *Guide for Care and Use of Laboratory Animals* published by the US National Institutes of Health (The National Academies Press, 8th Edition, 2011). The rats were anaesthetized by inhalation of isoflurane gas (70% N_2_O/30% O_2_). Then, the left external carotid artery was exposed and arterial branches were electro-coagulated. Balloon injury was created using an infiltrated 2F Fogarty balloon embolectomy catheter in the left common carotid artery as previously described^9^. A catheter was inserted through the transverse arteriotomy of the external carotid artery and positioned in 1 cm distance from a cut-down site. The catheter was then inflated and moved back and forth three times along the common carotid artery. For catheter-mediated intramural delivery of miRNA inhibitors, the transfection complex (200 nM miRNA/10 μl Lipofectamin RNAi_max_ in 200 μl Opti-MEM) were infused into the injured carotid lumen and then incubated for 15 min. For delivery of adenovirus, 2.5 × 10^8^ pfu virus in 100 μl PBS were infused into the injured lumen and then incubated for 20 min. The lumen was flushed once with saline before the catheter was removed. After the removal of catheter, the punched area was sealed and the clap in the common carotid artery was released to reestablish blood flow. Unless otherwise stated, the rats were then recovered in the cages for up to 10 days.

### Histopathological analysis

The balloon-injured rats were euthanized by inhalation of isoflurane according to the IACUC guideline and the common carotid arteries were excised after transcardiac perfusion-fixation with heparinized saline containing 3.7% formaldehyde^9^. The vessels were paraffin embedded and sectioned by rotary microtome (Leica RM2255). The two serial tissue sections (4 μm in thickness) were obtained from the middle area of common carotid arteries and stained with haematoxylin and eosin. The luminal, internal elastic laminal, and external elastic laminal areas were measured using NIH Image v 1.62. The intimal and medial areas were determined by subtraction of the luminal area from the internal elastic area and by subtraction of the internal elastic area from the external elastic area. The values from two serial sections per rat were averaged for analysis.

### RNA sequencing protocol

To perform small RNA and mRNA sequencing, total RNA was isolated from the rat carotid artery tissues using QIAzol Lysis Reagent (Qiagen) according to the manufacturer’s protocol. For sham control, we pooled three carotid tissues to increase the amount of total RNA. The purity and integrity of the total RNA extract was determined by NanoDrop 8000 spectrophotometer (Thermo Scientific) and Bioanalyzer (Agilent Technologies), respectively, and the samples with a RIN value greater than 8 were used for further sequencing process.

For small RNA sequencing, small RNA libraries were prepared using TruSeq Small RNA Prep kit (Illumina). Briefly, 1 μg of total RNA was ligated with 3ʹ and 5ʹ RNA adapters. Reverse transcription followed by PCR amplification were performed with primers that anneal to the 3ʹ and 5ʹ adapters for creating and enriching cDNA constructs. The cDNA library was purified with the Pippin prep electrophoresis platform (Sage Science). The quality of the library was verified by 2100 Bioanalyzer (Agilent Technologies). Small RNA sequencing was performed using Hiseq 2500 system (Illumina) with a 1 × 51 setup.

For mRNA sequencing, the mRNA sequencing libraries were prepared using TruSeq RNA Prep kit v2 (Illumina). Briefly, the mRNA samples were purified from 1 μg of total RNA using poly-T oligo-attached magnetic beads. The fragmented mRNAs were primed with random hexamers and were reverse transcribed. The mRNA templates were removed and the cDNA second strands were synthesized. After 3ʹ A-tailing and 5ʹ end repairing, DNA sequencing adaptors were ligated to the cDNA templates. Then, PCR were performed for amplifying the templates. The quality of the library was verified by 2100 Bioanalyzer (Agilent Technologies). The mRNA sequencing was performed using Hiseq 2500 system (Illumina) with a 2 × 101 setup.

### Sequencing data analysis

Fast QC was used for quality control followed by trimming adapter sequences with Cutadapt. For miRNA-Seq data, sequencing reads were mapped to the reference miRNA sequences in the miRBase release 19 using Bowtie V.0.12.9 with the perfect match option. The miRNA abundance was quantified using the quantile normalization method in R. The mRNA-Seq data were mapped to rat genome using STAR alignment tool. Transcript abundance was estimated at the gene level by RSEM (ver. 1.2.5).

Differentially expressed miRNAs (DEmiRNAs) were obtained edgeR (ver. 3.16.5), Voom (limmer 3.30.13), and DESeq2 (ver. 1.14.1), with the FDR cutoff < 10^–5^. We chose common miRNAs from three programs for reliable DEmiRNAs and further filtered out lowly expressed miRNAs with logCPM ≥ 3.

### Cell culture

Primary human aortic SMCs (HASMCs) were purchased from Lonza and expanded by sub-culturing in the Smooth Muscle Cell Growth Medium (SmGM) containing 5% fetal bovine serum with growth factors and antibiotics (cat. no. cc-4149, Lonza). HASMCs were mainly used for the experiments at passage number 5–7. HEK293T cells were cultured in DMEM containing 10% fetal bovine serum and 1% penicillin/streptomycin. All cultures were maintained in humidified incubator containing 5% CO_2_ at 37 °C.

The mirVana miRNA mimics or inhibitors (Invitrogen, USA) as well as siRNA (Bioneer, Korea) were transfected using Lipofectamine RNAi_max_ (Invitrogen). The cells were plated at a density of 1 × ${10}^{5}$ cells/well in a 6-well plate. At 24 h later, the transfection complexes containing either a miRNA mimic (0.1 nM each), miRNA inhibitor (10–150 nM each), or siRNA (100 nM each) were added to culture plate and then changed to fresh culture media after 24-h transfection.

### Real-time quantitative PCR

Total RNA was isolated from cultured HASMCs and rat carotid artery tissues using QIAzol Lysis Reagent (Qiagen) according to the manufacturer’s protocol. For the miRNA validation, individual cDNAs were synthesized from 40 ng of the isolated total RNA using TaqMan MicroRNA Reverse Transcription Kit and specific RT primers of TaqMan MicroRNA Assays (Applied Biosystems). Expression levels of mature miRNAs were quantified by quantitative real-time PCR using TaqMan Universal Master Mix II and specific FAM based probes and primers of TaqMan MicroRNA Assays (Applied Biosystems) according to the manufacturer’s protocol. The thermal cycling conditions for miRNAs were: 10 min at 95 °C for initial enzyme activation, followed by 40 cycles of 15 s at 95 °C (Denaturation) and 60 s at 60 °C (annealing/extension) for amplification. U87, snoRNA, and U6 were used as internal controls for rat carotid arteries. U6 was used as an internal control for HASMCs. For the mRNA validation, reverse transcription was performed from 1 μg of the isolated total RNA using ImProm-II RT system (Promega). Quantitative real-time PCR was performed using gene-specific primers (all Qiagen) and SYBR Green (Roche). The thermal cycling conditions for mRNAs were: 15 min at 95 °C for initial denaturation, followed by 40 cycles of 15 s at 94 °C (Denaturation) and 30 s at 55 °C (annealing) and 30 s at 72 °C (extension) for amplification. Melting curve analysis was carried out at the end of the PCR. The β-actin or 18S RNA was used as housekeeping gene. The CFX Connect Real-Time PCR Detection System (Bio-Rad) was used to detect levels of mature miRNAs and Transcripts. Relative gene expression was determined by ∆∆Ct value.

Information of the qPCR primers are described in the Supplementary Table S1.

### Immunoblot analysis

HASMCs were rinsed twice with ice-cold phosphate-buffered saline (PBS) and quickly frozen on liquid nitrogen^10^. Cells were lysed in lysis buffer containing 20 mM HEPES (pH 7.0), 1% Triton X-100, 150 mM NaCl, 10% glycerol, 1 mM EDTA (pH 8.0), 2 mM EGTA (pH 8.0), 1 mM DTT, 5 mM Na_3_VO_4_, 5 mM NaF, 1 mM AEBSF, 5 μg / ml aprotinin, and 5 μg / ml leupeptin. After centrifugation at 12,000×*g* for 10 min, the clarified lysates (30 μg each) were separated on denaturing polyacrylamide gels and transferred to nitrocellulose membranes. The membranes were incubated with primary antibodies in a Tris-buffered saline (TBS) solution containing 0.05% Tween-20 and 5% BSA for overnight at 4 °C. Next, the membranes were incubated with HRP-conjugated secondary antibodies (1:3000 dilution) in a TBS solution containing 0.05% Tween-20 and 5% skim milk for 1 h. The immune-reactive bands were visualized using WESTSAVE up ECL solution (cat. no. LF-QC0101, Abfrontier, Korea). Uncropped images for full-length blots are included in the Supplementary Information.

### Proliferation and cell cycle analysis

For cell proliferation assay, the miRNA inhibitor-transfected HASMCs were seeded at a density of 2000 cells/well on a 96-well plate, grown for the indicated times, and then incubated with a WST-1 reagent (10 μl/well) for 1 h at 37 °C. The number of viable cells was estimated by measuring the absorbance at 450 nm. Absorbance at 600 nm was subtracted as a reference wavelength. The assay was performed in duplicate.

For cell cycle analysis, HASMCs (1 × 10^5^ cells) were harvested after 48-h transfection. The cells were fixed and permeabilized in 70% ethanol for overnight at − 20 °C. The cells were treated with 100 μg/ml RNase A for 1 h at 37 °C and then stained with 10 μg/ml propidium iodide. The percentages of G_0_ / G_1_ diploid cells were analyzed by Modfit LT software (Verity Software House, Topsham, ME) in FACSCalibur system (BD biosciences).

### Immunofluorescence staining

For staining arterial tissues, the paraffin sections of balloon injured carotid arteries were de-paraffinized in xylene and rehydrated in ethanol^10^. The rehydrated tissue sections were boiled for 20 min in a citric acid-based antigen unmasking solution (Vector Laboratories) for antigen retrieval. For dual immunofluorescence, the tissue sections were blocked with 5% normal donkey serum in PBS-T (0.3% Triton X-100 in PBS) for 1 h. Then, the samples were incubated with FITC-conjugated anti-vWF antibody (1:50 dilution, Abcam) for overnight at 4 °C. After washing with PBS-T three times, the samples were incubated with Cy3-conjugated anti-SMA antibody (1:200 dilution, Sigma Aldrich) for 2 h at room temperature in the dark.

For the F-actin staining, HASMCs were fixed by 3.7% formaldehyde for 15 min and permeabilized by PBS-T for 15 min at room temperature. The cells were labelled with an Alexa Fluor 488-conjugated phalloidin (cat. no A12379, Invitrogen) for 60 min at room temperature.

For TUNEL assay, the fixed cells were incubated with a permeabilizaiton solution (0.1% Triton X-100, 0.1% sodium citrate) for 2 min at 4 °C. The cells were incubated with TUNEL reaction mixture for 60 min at 37 °C in the In Situ Cell Death Detection Kit (Roche Diagnostics).

Nuclear DNA was labeled with DAPI. Fluorescence images were obtained using LSM880 Airyscan confocal microscope (Carl Zeiss).

### Transwell migration assay

The chemotactic cell migration was measured using 24-well Transwell culture chambers (Costar; 8-mm pore size). The upper chamber was coated with gelatin B (1 mg/ml) and air-dried for 1 h. HASMCs were transfected with the miRNA inhibitors for 24 h, serum-starved for 18 h, and re-plated on the upper chambers with basal media at a density of 6000 cells/chamber. Complete media were added to the lower chambers. Transwell chambers were incubated at 37 °C for 24 h. The non-migrated cells were removed from the upper side of the membranes, and the cells passed through and attached to the lower side of the membranes were fixed and stained with 0.6% hematoxylin and 0.5% eosin. The number of stained cells from 4 different fields was counted and averaged.

### Monocyte adhesion assay

HASMCs were transfected with the miRNA inhibitors for 24 h and re-plated on a 96-well plate (4,000 cells/well). HASMCs were then stimulated with TNF-α (10 ng/ml) for 18 h. Separately, monocytic U937 cells (1 × ${10}^{6}$ cells/well) were stimulated with IFN-γ (50 μg/ml) for 24 h. The activated U937 cells were labeled with 4 μM tetramethylrhodamine ethyl ester, perchlorate (cat. No. T-669, Molecular Probes) for 30 min. The labeled U937 cells (1 × 10^5^ cells/well) were added to the confluent HASMCs and incubated at 37 °C for 1 h. The unbound U937 cells were gently removed by washing three times with PBS. The adherent U937 cells were detected using ZOE Fluorescent Cell Imager (Bio-Rad) and counted from two randomized fields.

### Luciferase reporter assay

A reporter plasmid, pMirTarget, containing the full-length 3’UTR sequence of human BMP-7 gene (hBMP-7–3ʹUTR) was purchased from Origene (cat. no. SC218118). For a negative control mutant, two consecutive nucleotides in the miR-370-3p target region #1 (nucleotide 229–235) of the hBMP-7–3ʹUTR were mutated (Supplementary Fig. S5C). For a reporter assay, HEK293T cells were plated on a 24-well plate and transfected with the reporter plasmid for 6 h. The cells were then transfected with a miR-370-3p mimic for 48 h and lysed for protein assay. The same quantity of cell lysates was subjected to the luciferase assay. The luminescence signal was measured in a VICTOR Multilabel Plate Reader (Perkin Elmer).

### In situ hybridization

Paraffin sections of human coronary arteries with atherosclerotic lesions were obtained from patients who underwent heart transplantations at Emory University Hospital, as approved by the Emory human subjects review committee^11^, and conducted following the guidelines in the Declaration of Helsinki. The informed written consent was given prior to the inclusion of subjects in the study. The miR-370-3p expression in the human coronary and rat carotid arterial tissues were detected by the miRCURY LNA miRNA in situ hybridization (ISH) Optimization Kits (Qiagen) according to the manufacturer’s protocol. The 5ʹ and 3ʹ DIG-modified has-miR-370-3p miRCURY LNA miRNA Detection Probe was used for hybridization. Briefly, 4-μm paraffin sections of the fixed arterial tissues were deparaffinized and rehydrated. Nuclease was inactivated by proteinase K for 10 min at 37 °C. The hybridization was performed by incubating with the miRNA detection probes (40 nM) for 1 h at annealing temperature (T_m_ − 30 °C). The slides were washed with a serial dilution of saline-sodium citrate (SSC) hybridization buffer at annealing temperature, immune-detection was performed by incubating alkaline phosphatase-conjugated anti-DIG antibody (cat. no. 11093274910, Roche) for 60 min at RT. For color development, the slides were then incubated with the NBT/BCIP (cat. no. 11697471001, Roche) substrate solution containing 0.2 nM Levamisol for 2 h at 37 °C. Cell nuclei were counterstained with Nuclear Fast Red (Sigma).

### Statistical analyses

Unless otherwise specified, data were analyzed by Student's *t*-test between two groups and one-way ANOVA with Turkey’s test for multiple groups. A *P* < 0.05 was considered to be statistically significant.

## Results

### Small RNA sequencing reveals a subset of miRNAs differentially expressed in the injured arteries

**Figure 1:**
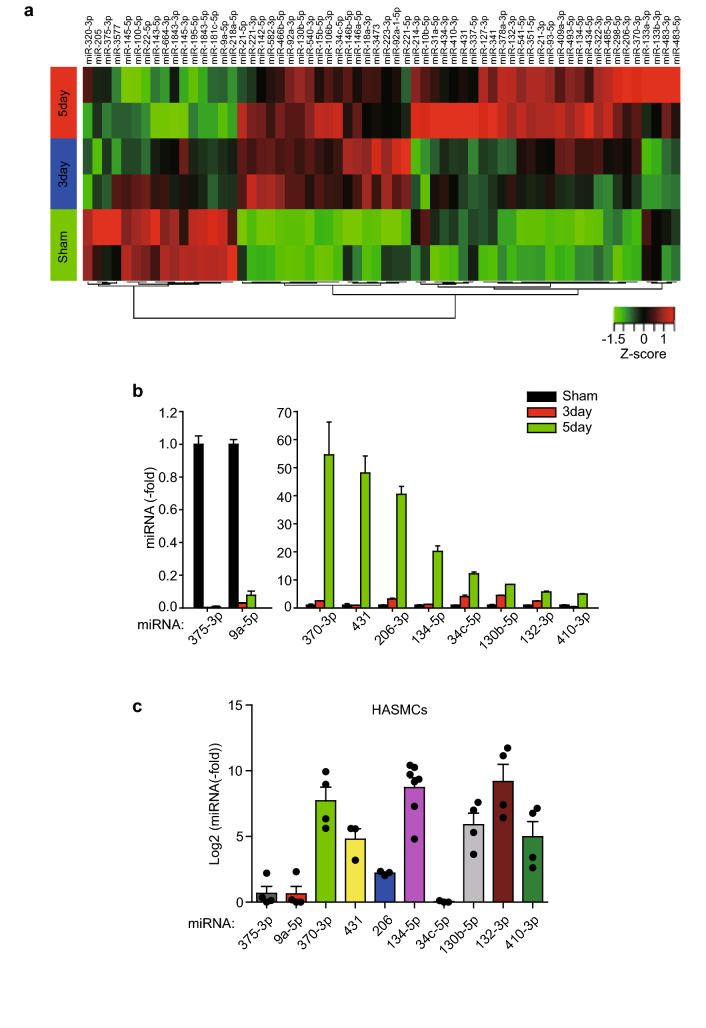
Profiling of miRNA expression at the early stage of the neointimal hyperplasia. (**a**) Heatmap depicting the relative expressions and correlations of the arterial 62 miRNAs identified by the small RNA sequencing in the balloon-injured rat carotid arteries (*n* = 2). (**b**) Relative expression levels of the rat arterial miRNAs. The carotid arteries were harvested at the indicated time points after balloon injury and pooled for miRNA-specific qPCR. Data in the graph are means ± SD of fold changes compared to sham control (*n* = 2). (**c**) Relative expression levels of the selected miRNAs in the cultured HASMCs. Data in graph are means ± SEM of the fold changes relative to the miR-375-3p level (*n* = 3–7).

We established the balloon injury model of rat carotid artery and examined the neointimal thickening over the injury time (Supplementary Fig. S1a). Based on the histological analysis, we chose two time points, 3- and 5-day post-balloon injury, to identify the miRNAs that direct the initiation of SMC dedifferentiation and proliferation. Subsequently, total RNA samples were prepared and separated into small RNAs and mRNAs by proper purification procedures. Both RNA pools were subjected to deep sequencing on the Illumina HiSeq2500 platform (Supplementary Fig. S1b). The small RNA sequencing data was analyzed to identify differentially expressed miRNAs (DEmiRNAs) with our in-house computational pipeline (Supplementary Fig. S2a). As shown in the heatmap (Fig. 1a), the 62 DEmiRNAs were identified at two consecutive time points compared to the control with sham operation. Interestingly, several miRNAs, such as miR-221-3p, miR-21-5p, and miR-146a-5p, have been known to be involved in the vascular SMC hyperplasia^8,12,13^, supporting the feasibility of our screening strategy using the rodent balloon injury model. Next, we performed quantitative verification by a miRNA-specific real-time PCR (Supplementary Fig. S2b) and selected 18 miRNAs showing expression change above fivefold for further functional characterization. In addition, some miRNAs, that are not identified in human database or have mismatched seed sequence between rats and humans were filtered out, leaving 10 miRNAs including 8 up-regulated and 2 down-regulated ones (Fig. 1b). To check the human relevance, we examined their expression level in the cultured primary human aortic SMCs (HASMCs) harboring a synthetic phenotype. The qPCR analysis indicated that six out of eight up-regulated miRNAs were prominently expressed in the HASMCs (Fig. 1c). Thus, our miRNA screening using a rodent model of neointimal hyperplasia discovered a subset of miRNAs with relevance to human vascular SMCs.

### Up-miRNAs regulate the SMC functions in cellular and in vivo model

**Figure 2:**
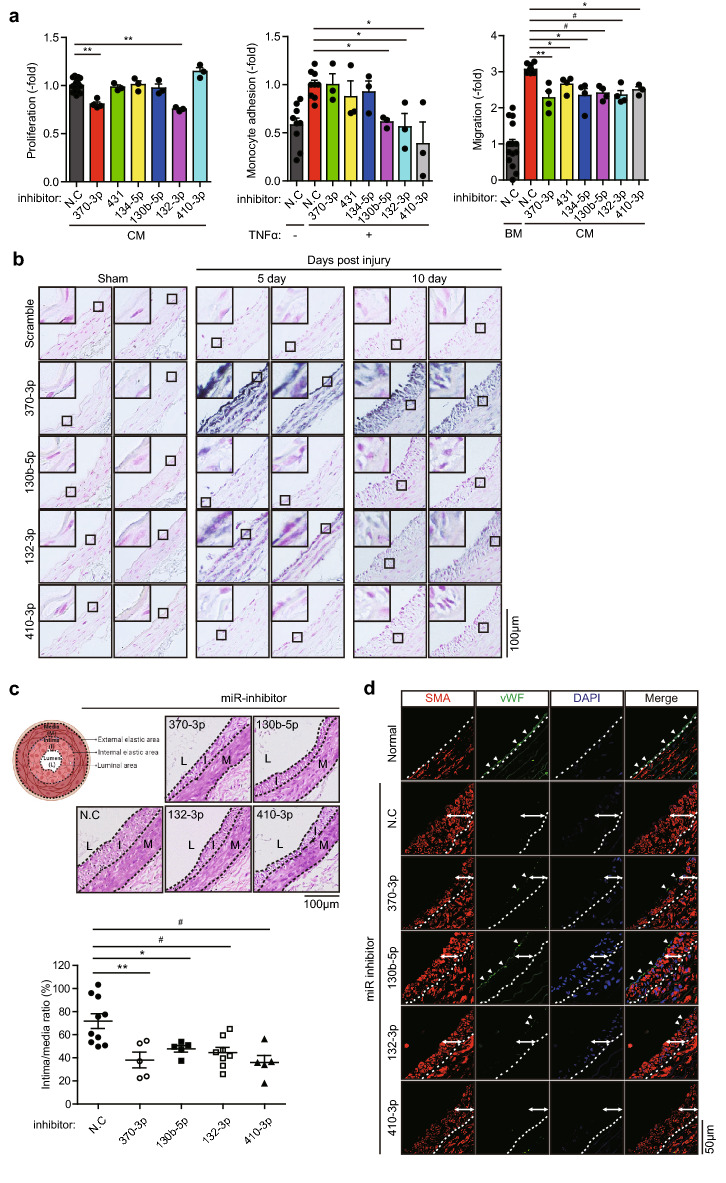
Expressions and inhibitory effects of the selected miRNAs on the neointimal hyperplasia. (**a**) Effects of the up-regulated miRNAs on the proliferation, migration, and monocyte adhesion of HASMCs. Cells were transfected with the indicated miRNA inhibitors for 24 h before each assay. For adhesion assay, HASMCs were stimulated with TNF-α (10 ng/ml). Data in the graphs are means ± SEM of relative fold changes versus non-specific control (N.C) (*n* = 3 or 4, *P < 0.05, **P < 0.005, ^#^*P* < 0.001). BM, basal medium; CM, complete medium. (**b**) In situ hybridization showing the high expression of each miRNA in the neointimal lesions of the rat carotid arteries. Nuclei were counterstained by nuclear fast red. Digoxigenin (DIG)-modified scrambled probe was used as a negative control. Representative images from two independent rats are shown. Scale bar 100 μm. (**c**) Effects of the miRNA inhibition on the neointimal hyperplasia in the injured rat carotids. The balloon-injured rat carotid vessels were infused with non-specific or specific miRNA inhibitors. Representative hematoxylin–eosin (HE) images in the tissue sections of carotid arteries harvested 10 days after injury. Data in the graph are means ± SEM of the intima-to-media ratio (n = 5–9, *P < 0.05, **P < 0.01, ^#^*P* < 0.005). A cartoon shows whole picture of the artery with labeling (L, lumen; I, intima; M, media). Scale bar 100 μm. (**d**) Effects of the miRNA inhibition on the arterial normalization. The balloon-injured carotid arteries were immunostained against von Willebrand factor (vWF) and α-smooth muscle actin (SMA). DAPI labels nuclei. The SMC and EC layers are indicated by arrows and arrowheads, respectively. Representative images are shown (*n* = 2). Scale bar 50 μm.

We evaluated the therapeutic application of the six up-regulated miRNAs that were prominently expressed in the HASMCs. To determine the role of these up-regulated miRNAs in the SMC functions, we conducted three types of cell-based assays in vitro using HASMCs transfected with miRNA inhibitors (Supplementary Fig. S3a). The SMC proliferation was significantly inhibited by miR-132-3p and miR-370-3p (Fig. 2a, 1st graph). As an inflammatory indication, the adhesion of monocytes to HASMCs was markedly inhibited by three miRNAs, namely miR-130b-5p, miR-132-3p, and miR-410-3p (Fig. 2a, 2nd graph). Interestingly, all six miRNAs tested significantly but slightly inhibited the SMC migratory activity (Fig. 2a, 3rd graph). According to these cell assays, we excluded two miRNAs (miR-134-5p and miR-431) possessing a marginal effect only on the SMC migration. Subsequently, we examined the effect of growth factors on the induction of the selected miRNAs. We chose platelet-derived growth factor (PDGF) and transforming growth factor-β (TGF-β) which play key roles in the SMC function^14,15^. Interestingly, PDGF induces all four miRNAs (miR-130b-5p, miR-132-3p, miR-370-3p, and miR-410-3p), whereas TGF-β slightly reduces only miR-132-3p, in the HASMCs (Supplementary Fig. S2c). This result indicates that the miRNAs selected from our deep sequencing might be effectors of growth factor signaling that regulates the SMC phenotype.

Subsequently, we evaluated the in vivo efficacy of the four selected miRNAs in the balloon-injured rat carotid model. First, we examined the arterial expression levels of these miRNAs by the in situ hybridization. The stained arterial tissue images showed that the expression of two proliferation-related miRNAs (miR-132-3p and miR-370-3p) was markedly increased in the balloon-injured carotid arteries compared with that in the sham-operated control (Fig. 2b). The two other inflammation-related miRNAs (miR-130b-5p and miR-410-3p) were also substantially induced by the balloon injury. Next, we evaluated the in vivo function of these four miRNAs in the balloon-injured rat arteries. Compared with that in the negative control, the catheter-mediated intramural delivery of the miRNA inhibitors markedly suppressed the neointimal hyperplasia in the balloon-injured lesion (Fig. 2c). Unexpectedly, the immunofluorescence staining of carotid arteries against von Willebrand factor and α-smooth muscle actin (SMA) as the EC and SMC markers, respectively, showed that the miR-130b-5p inhibitor facilitated the recovery of the EC monolayer, implying re-endothelialization (Fig. 2d). Thus, miR-130b-5p promoted the proliferation of SMCs but peculiarly prevented the proliferation of ECs. Overall, we verified that four miRNAs play the distinct roles, such as vascular inflammation and proliferation, in arterial homeostasis.

### Mitogenic miRNAs direct phenotypic change of VSMC

**Figure 3:**
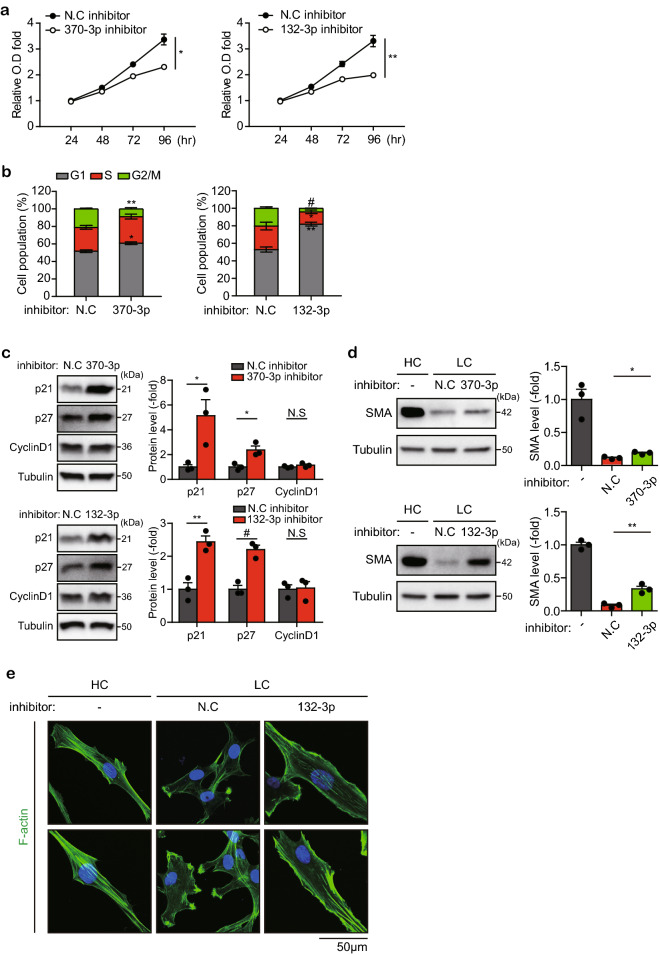
Roles of the selected DE-miRNAs in the SMC functions. (**a**) Effects of miR-370-3p or miR-132-3p on the proliferation rates of HASMCs. Data in the graphs are means ± SEM of the formazan absorbance at 450 nm after subtracting the absorbance at 600 nm as a reference (*n* = 3, *P < 0.05, **P < 0.005). (**b**) Effects of miR-132-3p and miR-370-3p on the cell cycle progression of HASMCs. Data in graphs are means ± SEM of the percentage of cells at each phase (*n* = 3, *P < 0.05, **P < 0.005, ^#^*P* < 0.001). (**c**) Effects of miR-132-3p and miR-370-3p on the expression of cell cycle proteins in HASMCs. Data in graphs are means ± SEM of relative fold changes of the band intensities normalized by the intensity of tubulin band (*n* = 3, *P < 0.05, **P < 0.01, ^#^*P* < 0.002). N.S., not significant. (**d**) Effects of miR-132-3p and miR-370-3p on the cell density-dependent phenotypic change of HASMCs. Highly-confluent (HC) culture of the miRNA inhibitor-transfected HASMCs were re-plated at a low confluence (LC, 8000 cells /cm^2^) and subjected to immunoblot analysis after 4-days culture. Data in graphs are means ± SEM of relative fold changes of the band intensities of α-smooth muscle actin (SMA) normalized by the tubulin intensity (*n* = 3, *P < 0.01, **P < 0.005). (**e**) Effects of miR-132-3p on the cell density-dependent cytoskeletal arrangements in HASMCs. The miRNA inhibitor-transfected HASMCs were stained with phalloidin (green). DAPI (blue) labels nuclei. Representative images are shown (*n* = 2).

In injured arterial tissues, the phenotype transition of SMCs from contractile to synthetic state by dedifferentiation precedes the proliferation-dependent neointimal hyperplasia. Therefore, we examined whether the proliferation-related miRNAs (miR-132-3p and miR-370-3p) can control the phenotype change. Both miRNA inhibitors induced cell cycle arrest at G1 phase, thereby suppressing the time-dependent proliferation of HASMCs (Fig. 3a,b). Consistently, both miRNA inhibitors markedly augmented the levels of two cyclin-dependent kinase inhibitors, p21 and p27 (Fig. 3c). However, the same miRNA inhibitors did not induce the apoptotic cell death of HASMCs (Supplementary Fig. S3b), indicating that these miRNAs indeed controlled the SMC proliferation. We next investigated the phenotype change in the HASMCs transfected with these miRNA inhibitors. The simplest way to mimic the arterial environment is to set the cell density in the culture^16,17^. Thus, we induced the contractile and synthetic phenotypes of HASMCs by plating cells at high and low densities, respectively, on the laminin-coated plate. The analyses of phenotype markers indicated that high and low cell densities clearly determined the contractile and synthetic SMC phenotypes, respectively (Supplementary Fig. S4a,b). Moreover, when the confluent SMCs were replated at a low density, they became gradually transformed into the synthetic phenotype, which was indicated by the reduced α-SMA levels (Supplementary Fig. S4c). Immunoblot analysis showed that the transfection of miR132-3p and miR-370-3p inhibitors restored the α-SMA level, which had been disappeared at a low cell density (Fig. 3d). Indeed, the immunofluorescence staining of F-actin filament demonstrated that low cell density induced the synthetic phenotype of HA SMCs and that the miR-132-3p inhibitor transformed these cells into the contractile phenotype (Fig. 3e). Thus, it is likely that miR-132-3p and miR-370-3p can regulate phenotype transition and proliferation signaling.

### Profiling target genes of the selected miRNAs

**Figure 4:**
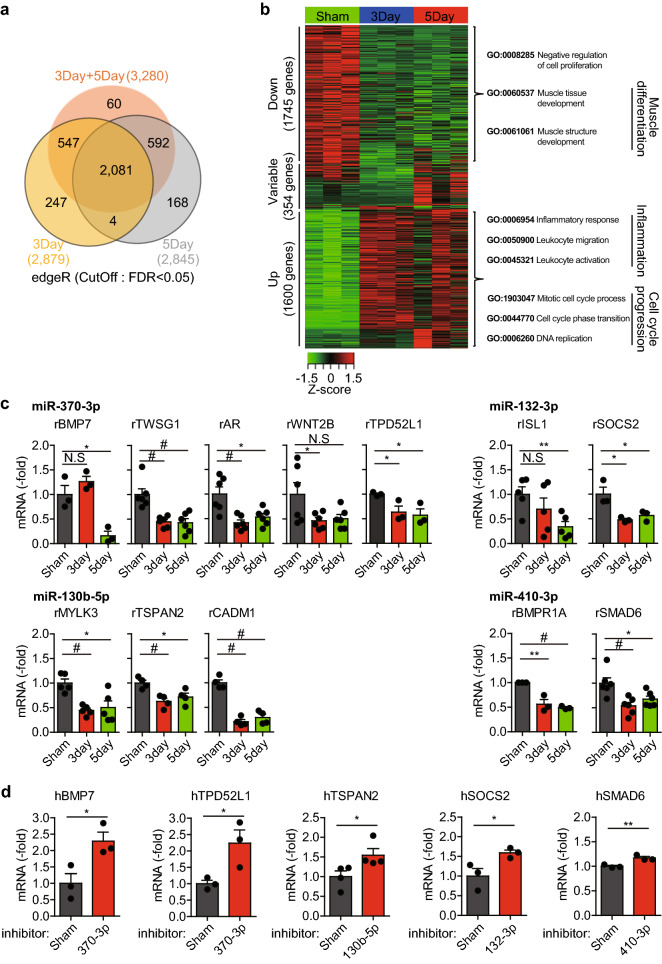
Identification of target genes specific to the selected miRNAs. (**a**) Venn diagram depicting the number of differentially-expressed genes (DEGs) overlapped between the recovery times post injury (*n* = 3, Cutoff: FDR < 0.05). (**b**) Heatmap depicting the relative expressions and correlations of the 3699 DEGs. (**c**) Expression of the miRNA target genes in the injured rat carotid arteries. Data in the graphs are means ± SEM of fold changes compared to sham control (*n* = 3–6, *P < 0.05, **P < 0.01, ^#^*P* < 0.005). (**d**) Expression of the miRNA target genes in the HASMC transfected with the indicated miRNA inhibitors. Data in the graphs are means ± SEM of fold changes compared to non-specific control (N.C) (*n* = 3–4, *P < 0.05, **P < 0.01).

To understand the biological consequences of the selected miRNAs, we next analyzed mRNA sequencing data from the rat balloon injury model. Differentially expressed gene (DEG) analysis with edgeR program (FDR < 0.05) identified 3699 DEGs that significantly changed expression at days 3 and/or 5 post-injury compared with those in the sham-operated control (Fig. 4a). The heatmap showed that the up-regulated and down-regulated DEGs were approximately half each (Fig. 4b). The gene ontology (GO) enrichment analysis further classified the DEGs into three functional clusters: muscle differentiation, inflammation, and cell cycle/proliferation. These clusters were associated well with cognate miRNAs. Using these DEGs, we then conducted in silico target profiling for the four selected miRNAs (miR-132-3p, miR-370-3p, miR-130b-5p, and miR-410-3p) in the target prediction databases (TargetScan, miRDB, and miRmap). The DEGs that were commonly predicted in both rat and human databases with high prediction scores (TargetScan < − 0.1, miRDB > 70, miRmap > 70) were considered as reliable targets of each miRNA. Subsequent real-time PCR highlighted that some DEGs were markedly down-regulated in the balloon-injured carotid arteries compared with those in the sham-operated control (Fig. 4c). To show the human relevance, we further examined the expression of the target genes in the HASMCs transfected with miRNA inhibitors. Consequently, the following real-time PCR revealed a key target gene for each miRNA, among which BMP-7, TSPAN2, SOCS2, and SMAD6 are specific targets for miR-370-3p, miR-130b-5p, miR-132-3p, and miR-410-3p, respectively (Fig. 4d).

### miR-370-3p/BMP-7 axis is essential for the SMC proliferation

**Figure 5:**
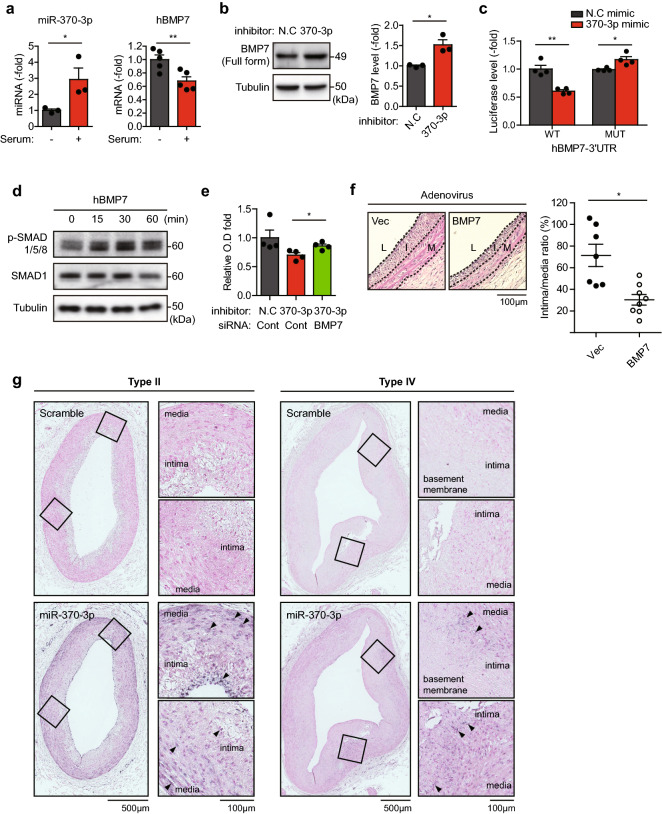
Direct inhibition of BMP-7 expression by miR-370-3p. (**a**) Comparison of miR-370-3p and BMP-7 expressions in the cultured HASMCs. HASMCs were left in the serum-free media or grown in the fetal bovine serum (10%)-containing media for 48 h. Data in graphs are means ± SEM of relative fold changes (*n* = 3–5, *P < 0.05, **P < 0.01). (**b**) Immunoblot analysis of the BMP-7 protein level in the HASMCs transfected with the miR-370-3p inhibitor. A representative immunoblot is shown. Data in the graph are means ± SEM of relative fold change (*n* = 3, *P < 0.05). (**c**) Effect of miR-370-3p on the expression of luciferase reporter linked to the 3ʹUTR of BMP-7 gene. HEK293T cells were serially-transfected with a firefly luciferase reporter construct containing human BMP-7–3ʹUTR region and a miRNA mimic in the indicated combination. Data in graph are means ± SEM of relative luciferase activity compared to non-specific control mimic (N.C) (*n* = 4, *P < 0.05, **P < 0.005). (**d**) BMP-7-induced R-SMAD phosphorylation in the HASMCs. The serum-starved SMCs were treated with human BMP-7 (1 ng/ml). Total SMAD was immunoblotted as a control. A representative immunoblot is shown (*n* = 2). (**e**) Effect of BMP-7 on the HASMC proliferation. HASMCs were co-transfected with both siRNA and miRNA inhibitor for 72 h and subjected to the WST-1 assay. Data in graph are means ± SEM of fold changes versus non-specific control (N.C) (*n* = 4, *P < 0.05). (**f**) Effects of the adenoviral expression of BMP-7 on the neointimal hyperplasia in the injured rat carotids. The balloon-injured rat carotid vessels were infused with adenoviruses encoding empty or human BMP-7 gene. Representative hematoxylin–eosin (HE) images in the tissue sections of carotid arteries harvested 10 days after injury. Data in the graph are means ± SEM of the intima-to-media ratio (*n* = 7–8, *P = 0.0025). L, lumen; I, intima; M, media. Scale bar 100 μm. (**g**) In situ hybridization showing the high expression of miR-370-3p in the neointimal lesions of coronary arteries obtained from human atherosclerotic patients (*filled arrowheads*). Type II and IV indicate the early and advanced disease grades, respectively. Nuclei were counterstained by nuclear fast red. Digoxigenin (DIG)-modified scrambled probe was used as a negative control. The lesion areas for magnification are indicated by rectangles. Scale bar 500 μm and 100 μm.

Because BMP family members play roles in the vascular function^18^, we deeply investigated the biological significance of BMP-7 as a miR-370-3p target in SMC proliferation. First, expressions of miR-370-3p and BMP-7 were validated in the HASMCs. Serum stimulation induced miR-370-3p expression but reduced BMP-7 expression (Fig. 5a). Consistently, western blot analysis demonstrated that miR-370-3p inhibition increased the protein level of BMP-7, which was validated by a knockdown experiment using human BMP-7-specific siRNA (Fig. 5b, Supplementary Fig. S5a). Furthermore, the miR-370-3p mimic evidently reduced the expression of luciferase reporter linked to the 3′-UTR of human BMP-7 gene, but not the reporter linked to the mutant 3′-UTR (Fig. 5c, Supplementary Fig. S5b). Taken together, BMP-7 is a true target gene of miR-370-3p in the HASMCs. Considering that recombinant BMP-7 treatment inhibits the SMC proliferation^19,20^, we assessed the involvement of miR-370-3p/BMP-7 axis in SMC proliferation. As a direct evidence, the BMP-7 treatment markedly induced the SMAD1/5/9 phosphorylation in HASMCs (Fig. 5d), suggesting an anti-mitogenic effect of BMP-7 on SMCs. Subsequent proliferation assay demonstrated that BMP-7 depletion restored the proliferation of SMCs, which were previously reduced by the miR-370 inhibitor (Fig. 5e). More strikingly, the adenoviral delivery of human BMP-7 gene to the balloon-injured carotid arteries strongly inhibited the neointimal hyperplasia compared to control virus (Fig. 5f).

Though present in the blood samples of patients with unstable angina, type II diabetes, and hyperlipidemia^21–23^, miR-370-3p has not been shown in the human arterial wall. Thus, we attempted to detect miR-370-3p in the human coronary arterial tissues with atherosclerotic lesion to find its correlation with vascular disease. Primarily, we validated the specificity of in situ hybridization by confirming the induction of miR-370-3p expression in HASMCs stimulated with serum (Supplementary Fig. S5c). The same in situ hybridization experiment demonstrated a higher expression of miR-370-3p in the coronary tissue sections from human patients with atherosclerotic type II and IV lesions (Fig. 5g), thereby supporting the idea that miR-370-3p is a novel miRNA relevant to atherosclerosis.

Collectively, the data represent that the miR-370-3p/BMP-7 axis is a crucial factor for SMC hyperplasia in injured arteries.

## Discussion

Given that miRNA has gained attention as a sophisticated tool for modulating gene expression, considerable research efforts have been exerted in the vascular biology field. The first screening study was conducted in 2007, using an early type of microarray containing only 140 miRNA probes^8^. Recently, few disease-focused screening studies identified a group of miRNAs that play a role in the phenotypic change of vascular SMCs^6,7^. Many miRNAs have also been found in the vascular SMCs and ECs concerning arterial injury^24^. However, no specific miRNA-targeted therapeutics have been clinically available for vascular therapy^25^. Hence, we envisioned an alternative deep sequencing-based miRNA screening strategy by combining the rat carotid injury model and human cell-based validation. Through this strategy, we identified a new subset of miRNAs and some of them have not yet been described in the human arterial SMC type.

**Figure 6:**
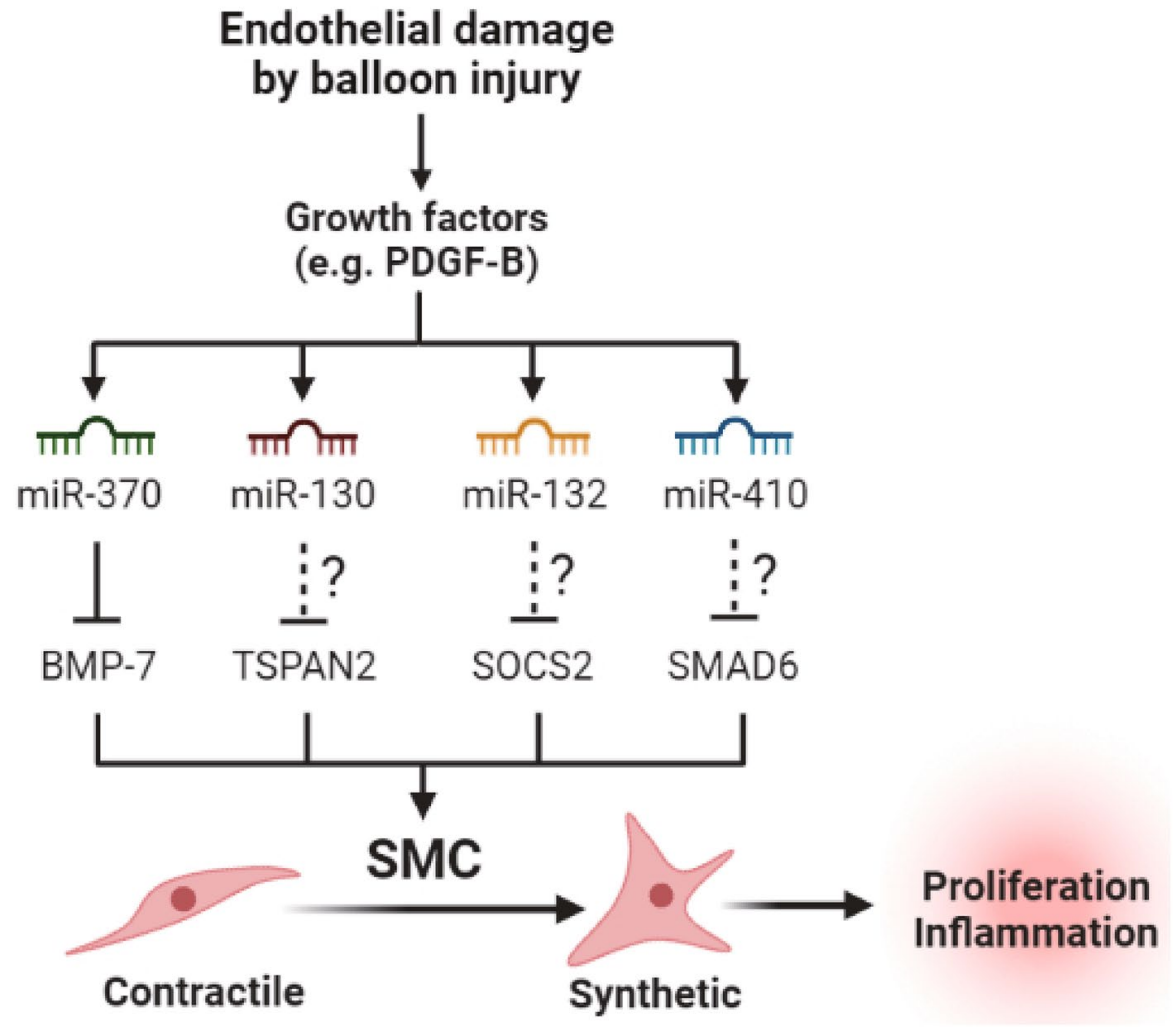
Model of the miRNA-mediated regulation of SMC phenotypes. Endothelial damage by arterial injury triggers growth factor-mediated induction of four miR candidates in the arterial SMCs. Those miR candidates suppress expression of their particular target genes and trigger the phenotype change of SMCs from contractile to pathogenic synthetic type.

Simultaneous profiling of miRNA and mRNA transcriptomes successfully revealed the role of these miRNAs in the inflammatory response and phenotypic change of vascular SMCs (Fig. 6). In this study, we found that miR-370-3p repressed the gene expression of BMP-7. Although the function of BMP-7 is not well-characterized in the arterial wall, an in vitro evidence demonstrated that BMP-7 counteracts the TGF-β signaling that stimulates SMC proliferation^20^. Indeed, we showed an in vivo evidence that adenoviral expression of BMP-7 targeted to the balloon-injured carotid artery suppresses the intimal hyperplasia. Thus, it is likely that miR-370-3p/BMP-7 axis can be a good target for treating vascular diseases harboring SMC hyperplasia. Besides, many studies also indicated the relevance of other miR targets to inflammation and atherosclerosis. For example, TSPAN2 suppresses SMC proliferation and its low expression is observed in the atherosclerotic lesion^26^. SOCS2 is known to inhibit inflammatory response in ECs and monocytes^27,28^. SMAD6 is involved in the EC homeostasis^29^.

Unexpectedly, among the up-regulated miRNA candidates miR-130b-5p was involved in the destruction of endothelial monolayer in the balloon-injured carotid arterial wall. Previously, miR-21 and miR-221/222 have also been shown to reciprocally regulate the proliferation and migration of SMCs and ECs^24^. Interestingly, a recent study reported that level of miR130b-5p was detected in the peripheral blood of patients with coronary artery disease and negatively correlated the disease severity^30^. Thus, the effect of this miRNA in the endothelial integrity remains to be further inspected.

## Supplementary Information

Supplementary Information.

## Data Availability

The RNA sequencing data underlying this article are available in ArrayExpress (http://www.ebi.ac.uk/arrayexpress), under accession no. E-MTAB-11057 and E-MTAB-11100. The data that support the findings of this study are available from the corresponding author upon reasonable request. The authors declare that all the data supporting the findings of this study are available within the paper and supplementary information files.
