# Supplementary material for: Conserved miR-370-3p/BMP-7 axis regulates the phenotypic change of human vascular smooth muscle cells
Source: Sci Rep. 2023 Feb 10;13:2404. doi: 10.1038/s41598-022-26711-z (PMC9918535; doi:10.1038/s41598-022-26711-z)
Supplement: Supplementary file 1 — Supplementary Information. [file 41598_2022_26711_MOESM1_ESM.pdf]

Supplementary Table S1. Information of the qPCR primers

A. Primers for miRNAs

miRNA	Cat. No. (Applied Biosystems)	Assay ID
miR-375-3p	4427975	000564
miR-9a-5p	4427975	000583
miR-370-3p	4427975	002275
miR-206	4427975	000510
miR-134-5p	4427975	001186
miR-34c-5p	4427975	000428
miR-130b-5p	4427975	002114
miR-132-3p	4427975	000457
miR-410-3p	4427975	001274
U87	4427975	001712
snoRNA	4427975	001718
U6	4427975	001973

B. Primers for mRNAs

mRNA (Rat)	Cat. No. (Qiagen)	GeneGlobe ID
BMP7	249900	QT01620885
TWSG1	249900	QT01616825
AR	249900	QT00184394
WNT2B	249900	QT00396144
TPD52L1	249900	QT01317603
MYLK3	249900	QT01566656
TSPAN2	249900	QT00182182
CADM1	249900	QT01619429
ISL1	249900	QT00188776
SOCS2	249900	QT00384629
BMPR1A	249900	QT00193165
SMAD6	249900	QT01582175

mRNA (Human)	Cat. No. (Qiagen)	GeneGlobe ID
BMP7	330001	PPH00527A-200
TPD52L1	330001	PPH08107A-200
TSPAN2	330001	PPH14756A-200
SOCS2	330001	PPH00756B-200
SMAD6	330001	PPH01910F-200

mRNA	Sequence (Sense)	Sequence (Antisense)
KLF4	TCAGTGGCCGCCACCGTGTC	GTGAAGCTGCAGGTGGAGGGC
Vimentin	GCGAGGAGAGCAGGATTTCTC	ACCAGAGGGAGTGAATCCAGA
18S	GGCCGTTCTTAGTTGGTGGAGCG	CTGAACGCCACTTGTCCCTC
β-Actin	TGGATCAGCAAGCAGGAGTATG	GCATTTGCGGTGGCAGAT

Conserved miR-370-3p/BMP-7 axis regulates the phenotypic change of human vascular smooth muscle cells.

Yerin Kim, Namhee Yu, Ye Eun Jang, Eunkyung Lee, Yeunjoo Jung, Doo Jae Lee, W. Robert Taylor, Hanjoong Jo, Jaesang Kim, Sanghyuk Lee*, Sang Won Kang*

Supplementary Figures

Figure S1

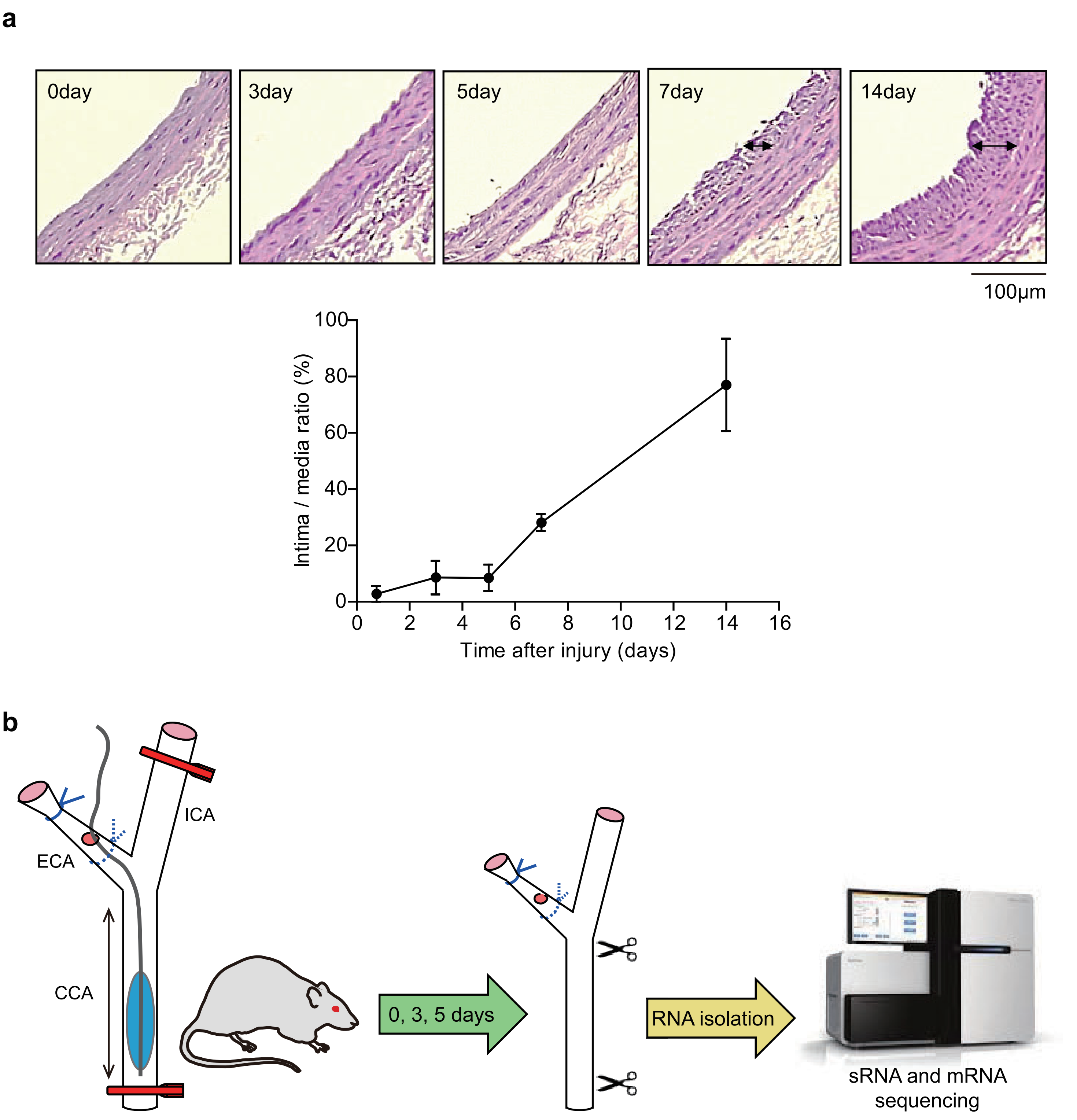

Fig. S1 RNA sequencing procedure in the balloon-injured rat carotid model.
a. Neointimal thickening in the balloon-injured rat carotid arteries. Data in the graph are means ± SEM of the intima-to-media ratio (n = 3). Representative images are shown. Scale bar: 500 μm.
b. Schematic drawing for the RNA sample preparation and sequencing procedure. The blue solid and dotted lines indicate permanent ligation before and after the injury, respectively.
The red lines indicate temporal clamping.

Figure S2

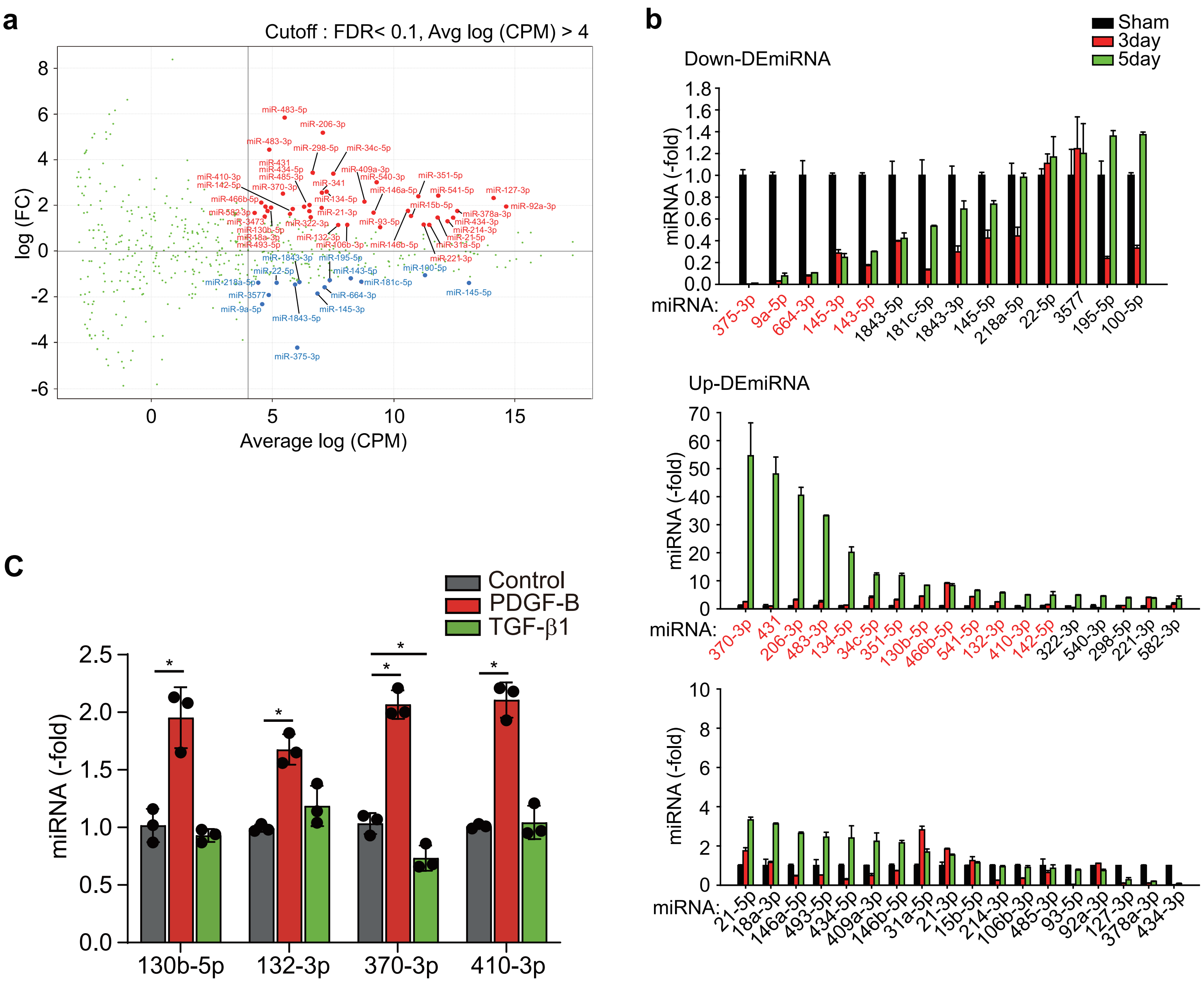

Fig. S2 Profiling of differentially-expressed miRNAs in the rat carotid arteries subjected to the balloon injury.
a. The MA plot of the log2 fold changes (log FC) versus the log2 average expression (Average log CPM) of the differentially-expressed miRNAs. The up-regulated and down-regulated miRNAs with significant changes (FDR < 0.1 for log(FC); Average log(CPM)>4) are indicated by red and blue colors, respectively.
b. Real time qPCR data showing the relative expression levels of the DE-miRNAs. Data in graphs are means ± SD of fold changes relative to sham control. The selected miRNAs are indicated by red color.
c. Expression levels of miRNAs in the HASMCs treated with growth factors. HASMCs were serum-starved for 24 h and stimulated with PDGF-BB (50 ng/ml) or TGF-β1 (2.5 ng/ml) for 24 h. Data in graphs are means ± SD of fold changes relative to untreated control (*n* = 3, *P < 0.01, **P < 0.001).

Figure S3

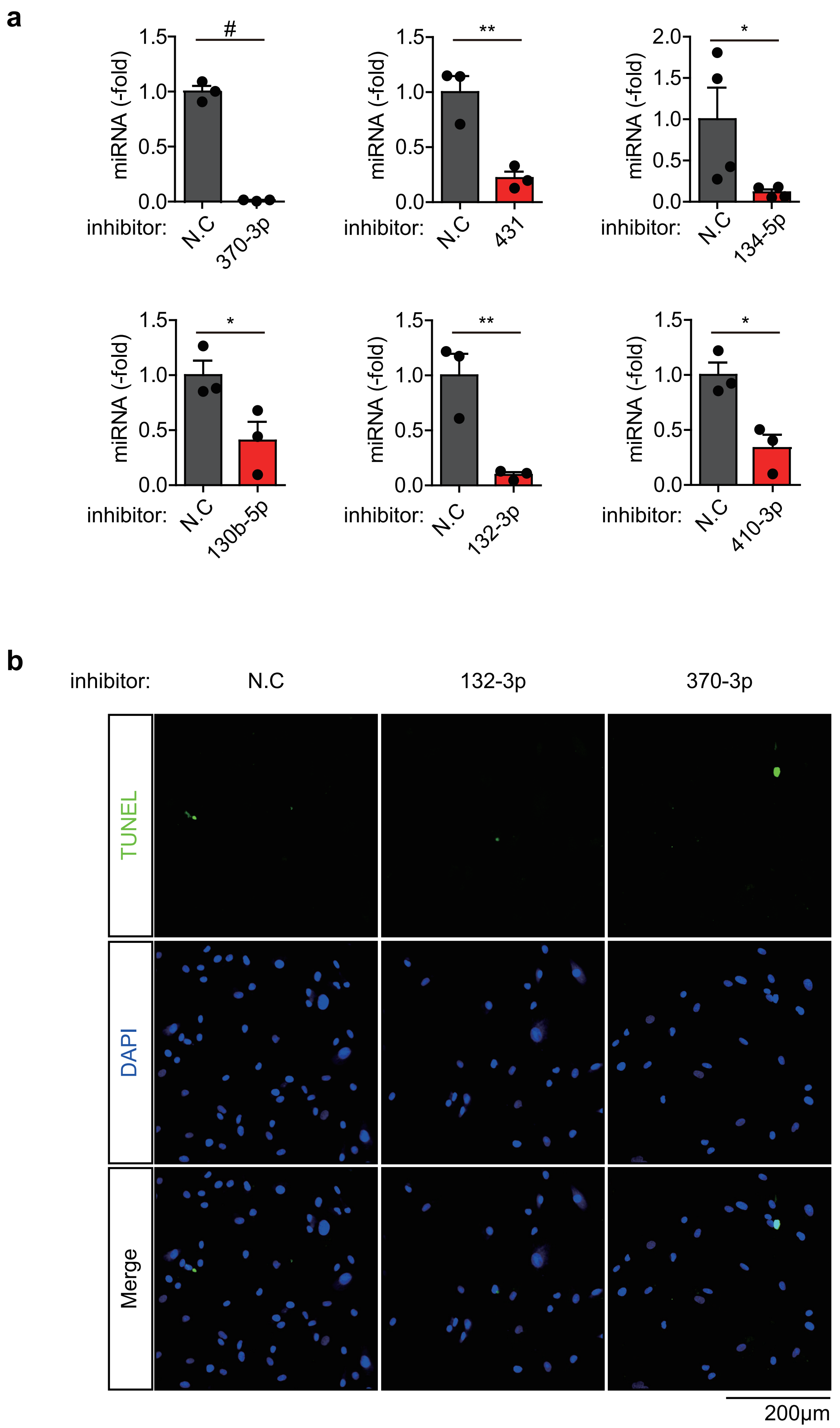

Fig. S3 Effect of miRNA inhibitors on SMC apoptosis.

a. Knockdown of miRNA expressions by specific miRNA inhibitors in the HASMCs. HASMCs were transfected with each miRNA inhibitor for 48 h. Data in graphs are means ± SEM of fold changes compared to non-specific control (N.C) (n = 3 or 4 , *P < 0.05, **P < 0.01, #P < 0.005).

b. Effects of miR-132-3p and miR-370-3p on the SMC apoptosis. Apoptotic cells were identified by TUNEL staining (green). DAPI labels nuclei (blue). No apoptotic cell were detected at 72 h after transfecting with miRNA inhibitors. Scale bar: 200 μm.

Figure S4

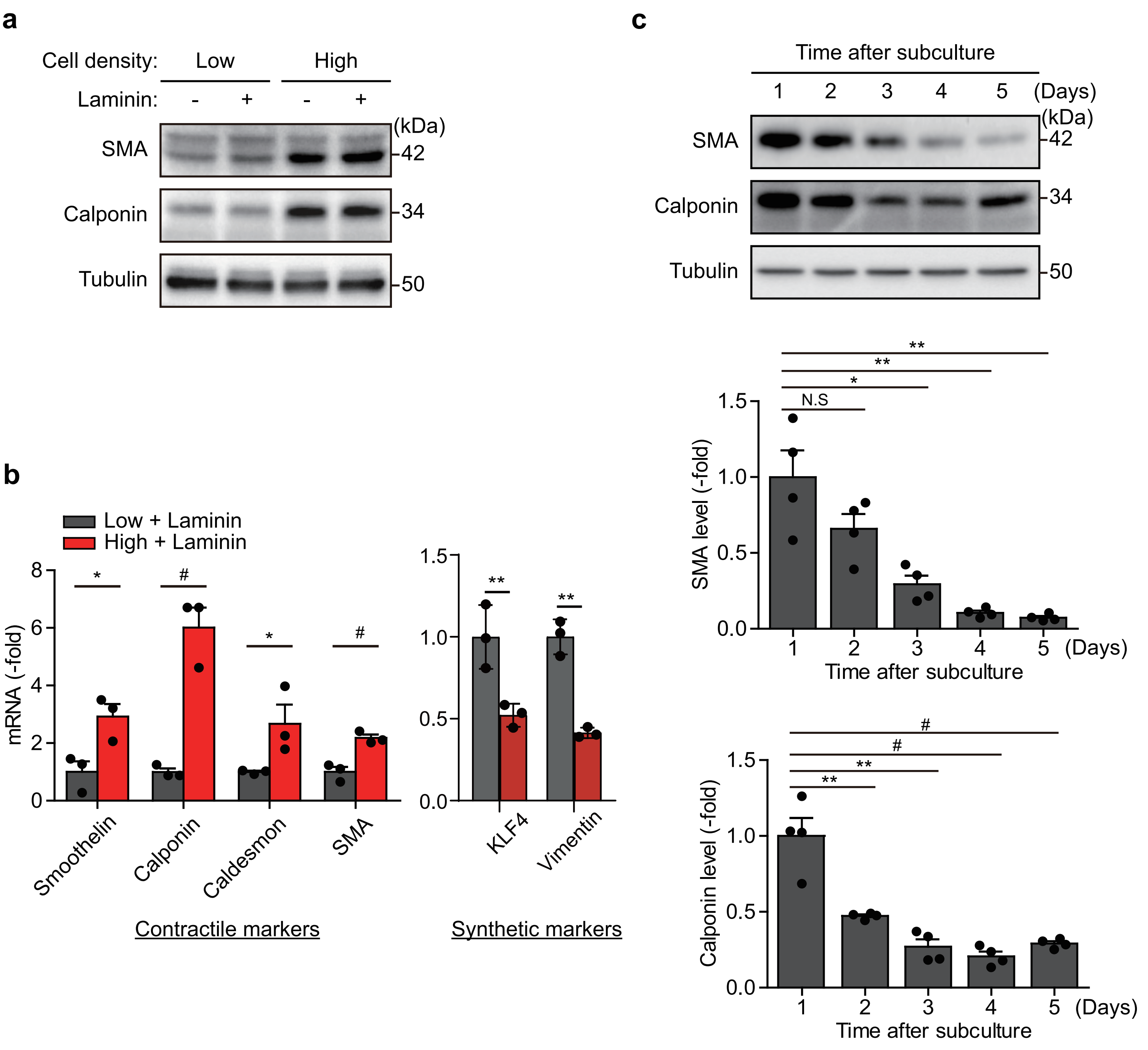

Fig. S4 Cell density-dependent phenotypic switch in HASMCs.
a and **b**. Cell density-dependent SMC phenotypes. HASMCs were seeded at either low density (8×10^3 cells/cm^2) or high density (1×10^6 cells/cm^2). The culture plates were remained uncoated or coated with an extracellular matrix laminin (10 μg/ml). Levels of contractile and synthetic phenotype markers were measured by immunoblotting (**a**) and real-time qPCR (**b**). Data in graphs (**b**) are means ± SEM of fold changes relative to low-density cells (n = 3, *$P < 0.05$, **$P < 0.01$, $^{\#}P < 0.005$).
c. Induction of SMC transition from contractile to synthetic phenotypes by cell density. The high-confluent HASMCs were re-plated at low density and analyzed by immunoblotting the contractile markers at the indicated times. Data in graphs are means ± SEM of fold changes relative to the band intensity in the first lane (n = 4, *$P < 0.01$, **$P < 0.005$, $^{\#}P < 0.001$). N.S., not significant.

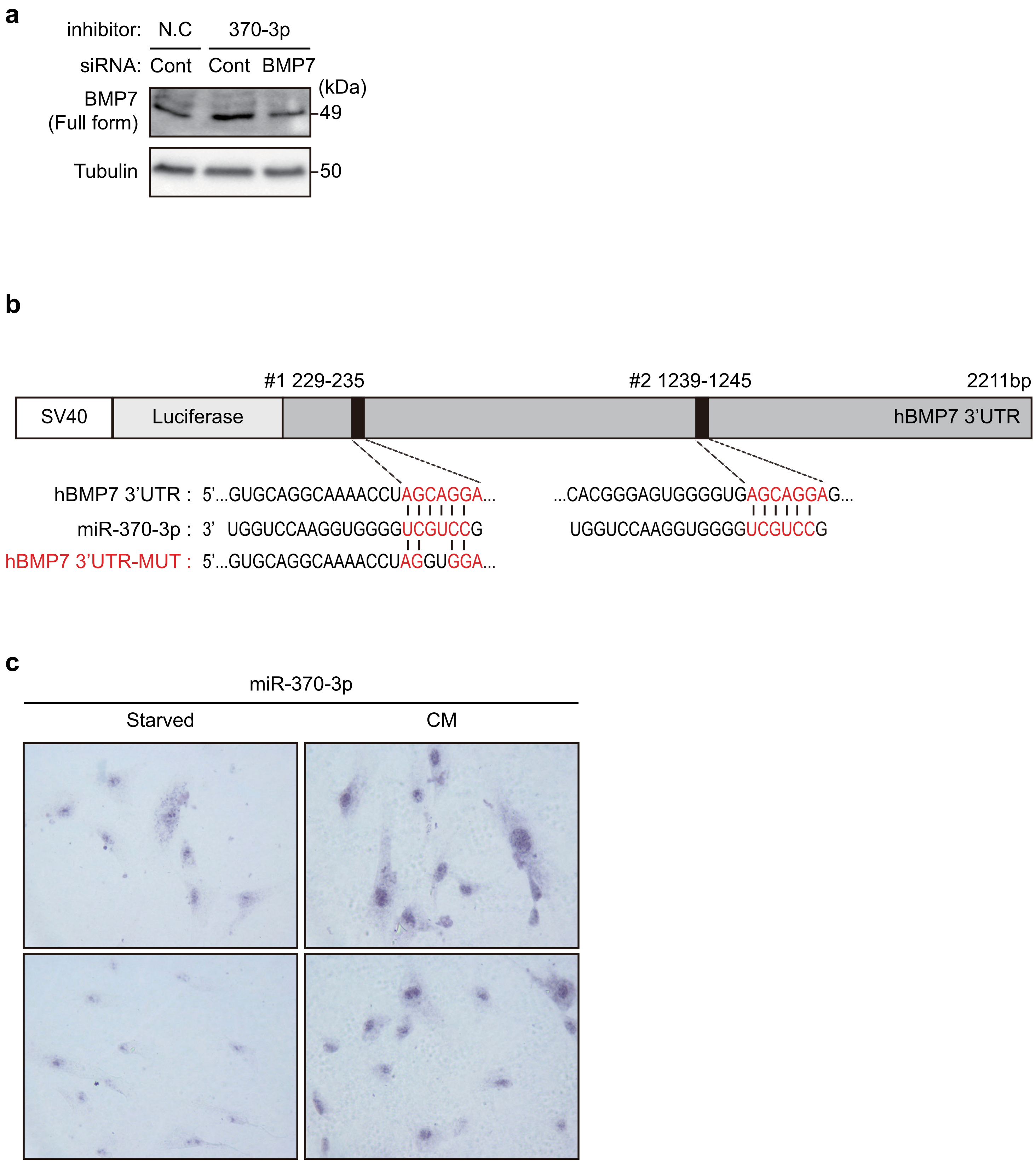

Fig. S5 MiR-370-3p regulates the BMP7 expression.
a. Expression of BMP7 protein in the HASMCs transfected with miR-370 inhibitor. A siRNA specific to human BMP7 was co-transfected with miRNA inhibitor for 48 h. A representative immunoblot is shown.
b. Schematic drawing of luciferase reporter construct containing the 3' UTR of human BMP7 gene. Two potential target sites of miR-370-3p on 3 UTR of human BMP7 gene were predicted by Targetscan 7.2 and miRmap database. The wild-type and mutant target sequences are illustrated.
c. Serum-induced expression of miR-370-3p in HASMCs. The level of miR-370-3p was analyzed by in situ hybridization in the serum-starved or serum-treated HASMCs. Images from two independent experiments are shown.

[Uncropped immunoblot images]

Fig. 3c

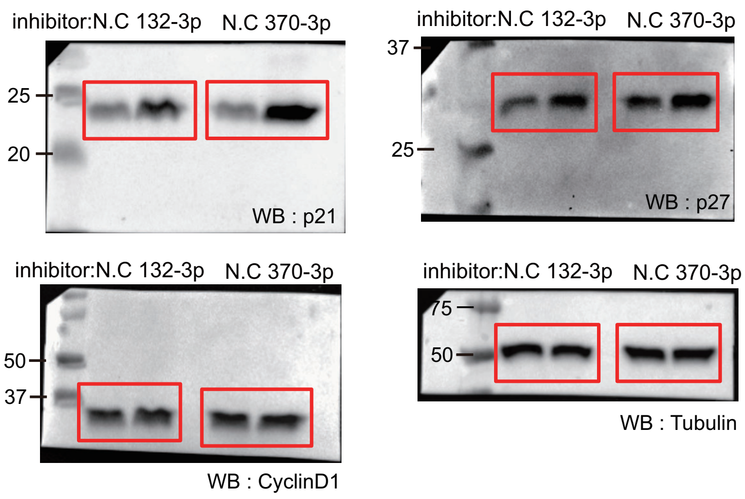

Fig. 3d

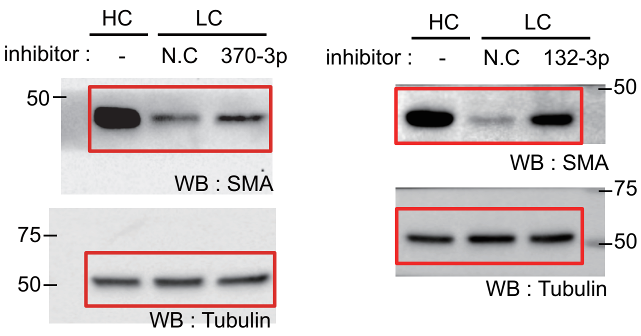

Fig. S4a

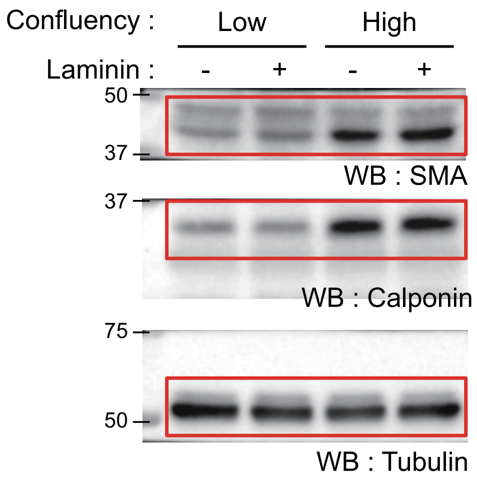

Fig. S4c

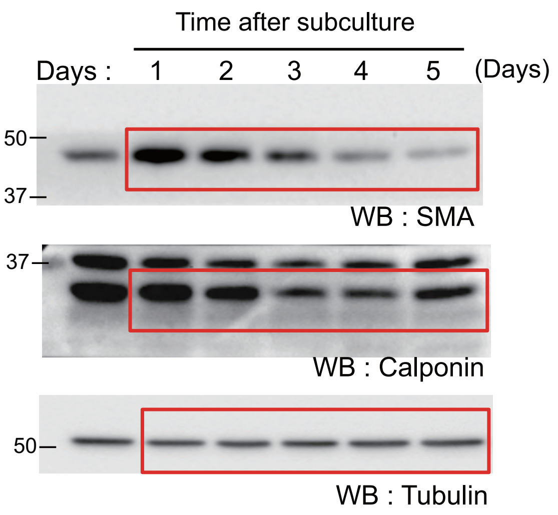

Fig. S5a

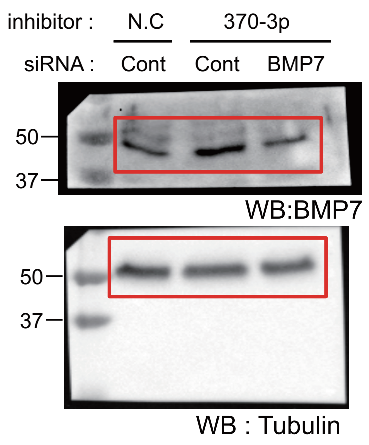

Fig. 5b

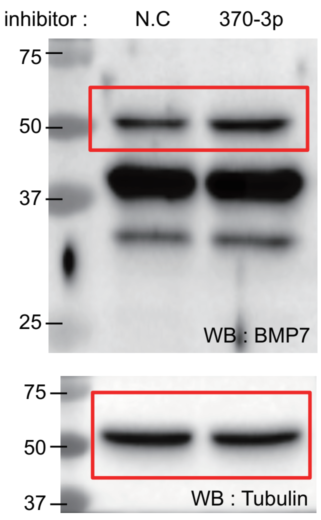

Fig. 5d

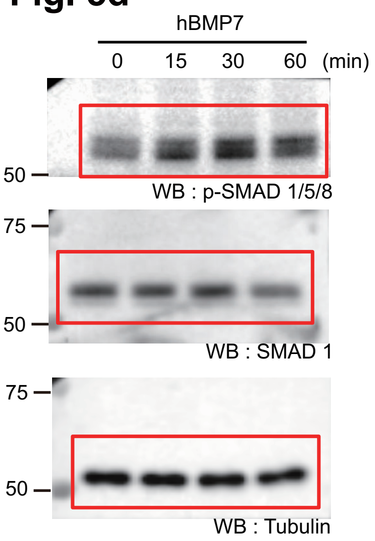

This images were taken using a ChemiDoc station and show the full-length membranes.
Some membranes were cut for hybridization with different antibodies.
Red boxes indicate the cropped region on the membrane and used in the figures.
